# Supplementary material for: DNA Methylation at a Single Locus of Human Genome Accurately Recapitulates Episignature of CREBBP-Related Rubinstein–Taybi Syndrome
Source: Int J Mol Sci. 2025 Sep 19;26(18):9183. doi: 10.3390/ijms26189183 (PMC12470928; doi:10.3390/ijms26189183)
Supplement: Supplementary file 1 [file ijms-26-09183-s001.zip › Supplementary Materials 2.pdf]

$$\text{median}\ (x_i) \tag{S1}$$

$$\text{scale} = \frac{100\%}{median\ (xi)} \tag{S2}$$

$$k = \text{scale}*b \tag{S3}$$

$$b = 100\% - k, \tag{S4}$$

where x_i is a vector of the signal level at the nucleotide *i* in amplicons with no methyl groups, scale is the correction vector for each nucleotide, k is the corrected signal level, b is the signal level of each nucleotide in the studied samples.

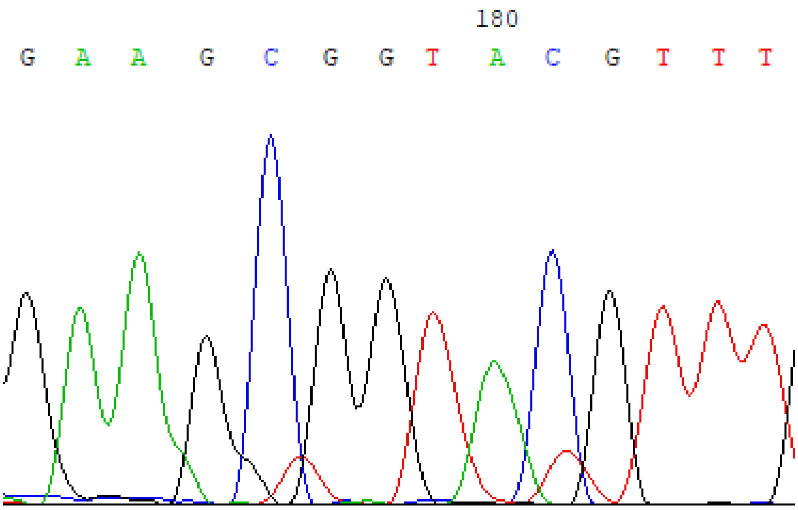

(a)

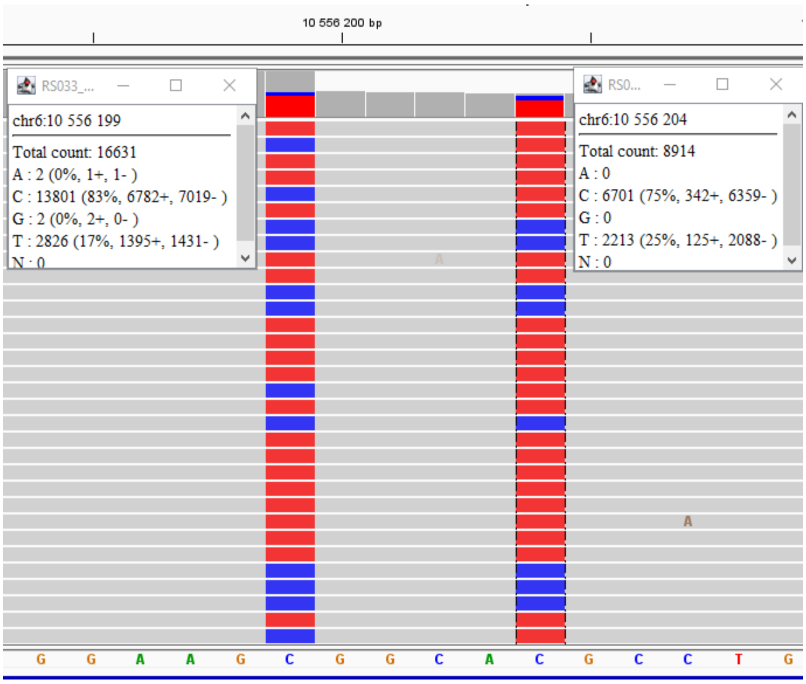

(b)

Supplementary Figure S1. Sample sequence of the selected locus for a patient with RTS1. (**a**) Sanger bisulfite sequencing (**b**) Targeted high-throughput bisulfite sequencing.

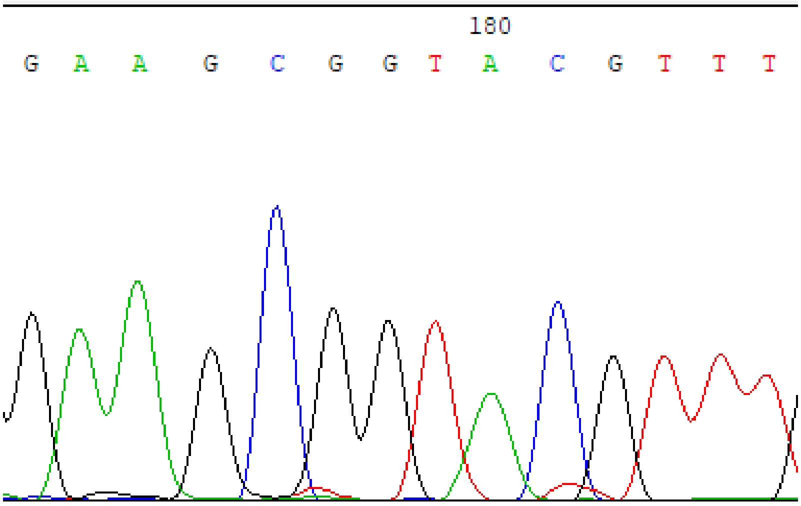

(**a**)

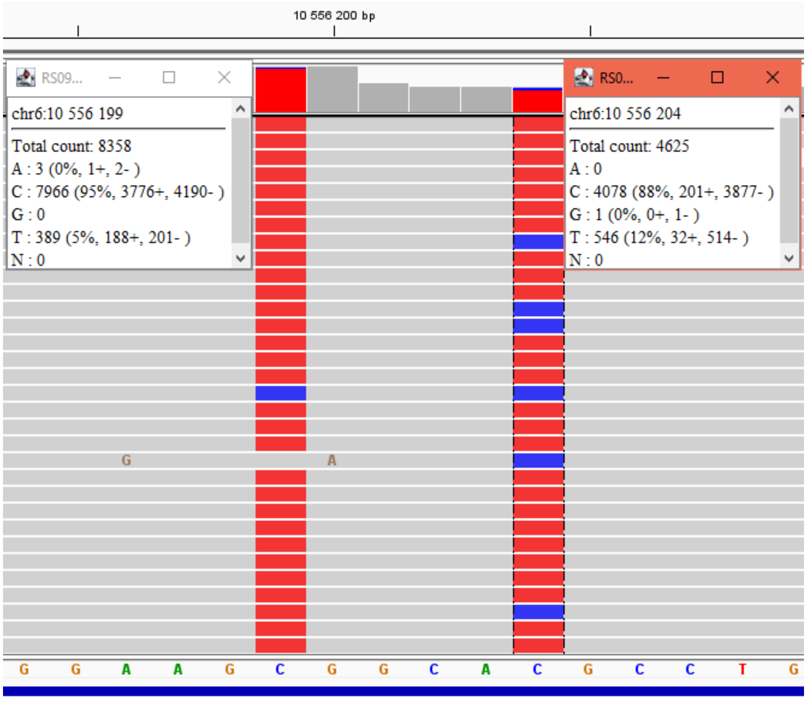

(**b**)

Supplementary Figure S2. Sample sequence of the selected locus for a healthy control. (**a**) Sanger bisulfite sequencing (**b**) Targeted high-throughput bisulfite sequencing.
